# Congruency effects can interact even when controlling for the reactivation aversion effect: implications for the generality-specificity paradox in hybrid distractor-interference tasks

**DOI:** 10.1007/s00426-025-02216-y

**Published:** 2025-12-12

**Authors:** Matthew G. Dunaway, Daniel H. Weissman

**Affiliations:** Department of Psychology, University of Michigan, 530 Church St, Ann Arbor, MI 48109 USA

## Abstract

Hybrid distractor-interference tasks frequently produce a paradoxical data pattern. Within-trial congruency effects associated with two distractors interact, consistent with domain-general control, while across-trial congruency sequence effects (CSEs) associated with the same distractors sum additively, consistent with domain-specific control. Recent findings from the hybrid prime-Simon task suggest that controlling for a confound called the reactivation aversion effect (RAE) resolves this “generality-specificity paradox” by eliminating the within-trial interaction between congruency effects. As there are competing accounts of this interaction, however, it remains unclear whether controlling for the RAE always eliminates it. To contrast this possibility with the competing accounts, we varied across three experiments (*N* = 168) (a) whether one of the two congruency effects was relatively large or relatively small and (b) whether two distractors engendered the same or different types of conflict. We observed a within-trial interaction while controlling for the RAE when one of the two congruency effects was relatively large regardless of whether the two distractors engendered the same or different types of conflict. This outcome suggests that controlling for the RAE may not always resolve the generality-specificity paradox. It also supports the view that observing a within-trial interaction is more likely when there is sufficient “operating space” for congruency effects to interact.

## Introduction

Distractions are abundant and consequential in everyday life. For example, imagine waiting behind a row of red lights for your turn to drive through a crowded intersection. Suddenly the light corresponding to an adjacent lane turns green and you feel yourself taking your foot off the brake. In this situation, the green signal is a distraction that might cause you to unsafely enter the intersection. To minimize the potentially adverse consequences of such behaviors (e.g., traffic accidents), it is crucial to investigate how people cope with distraction.

In the laboratory, researchers use distractor-interference (e.g., Stroop, flanker, Simon) tasks to investigate this ability (Eriksen & Eriksen, 1974; Simon, 1969; Stroop, 1935). In such tasks, participants identify a target while attempting to resist the influence of one or more distractors. For example, in the Stroop task participants identify the ink color (target) in which a color word (distractor) is presented (Stroop, 1935). Participants are slower and more error prone in incongruent trials wherein the word and the ink color do not match (e.g., RED in green ink) than in congruent trials wherein the word and the ink color do match (e.g., RED in red ink). This difference in reaction time and/or error rate is an experimental index of distraction known as the congruency effect and suggests that people are unable to completely ignore irrelevant stimuli.

Another frequent finding in distractor-interference tasks is that the congruency effect is smaller following incongruent trials than following congruent trials (Gratton et al., 1992). This “congruency sequence effect” (CSE) is consistent with the view that control processes adapt to distractions in ways that minimize the influence of future distractions on performance. For example, control processes may shift attention away from the distractor and toward the target after incongruent trials, thereby reducing the congruency effect (Botvinick et al., 2001; Gratton et al., 1992). In some tasks, CSEs reflect feature integration (i.e., stimulus-response repetition) confounds (Hommel et al., 2004; Mayr, 2003) and/or contingency learning (i.e., stimulus-response frequency) confounds (Schmidt & De Houwer, 2011). Robust CSEs, however, can appear even without these prevalent confounds consistent with a contribution of control processes to this phenomenon (Kim & Cho, 2014; Schmidt & Weissman, 2014; Weissman et al., 2014).

### Hybrid distractor-interference tasks

Researchers use *hybrid* distractor-interference tasks to investigate how control processes minimize distraction from multiple distractors at the same time (e.g., Egner, 2008; Hommel, 1997; Kornblum, 1994; Rey-Mermet, 2020; Weissman, 2020). These tasks include at least two distractors (e.g., flanker *and* Simon). Further, the congruency of each distractor (e.g., congruent or incongruent) varies independently of the congruency of every other distractor. In the hybrid flanker-Simon task, for example, one can classify each trial jointly in terms of its flanker congruency and Simon congruency as follows: (1) both congruent, (2) flanker-congruent/Simon-incongruent, (3) flanker-incongruent/Simon-congruent, or (4) both incongruent.

Data from hybrid distractor-interference tasks frequently produce a puzzling pattern. The within-trial congruency effects that are associated with each of two distractors often interact, even though the across-trial CSEs associated with the same distractors sum additively (Rey-Mermet, 2020; Schlaghecken et al., 2011; Schlaghecken & Maylor, 2020). More specifically, the within-trial congruency effect associated with either distractor is smaller when the other distractor is incongruent as compared to congruent, even though the across-trial CSE associated with either distractor does not vary with the congruency of the other distractor in the previous trial. Some researchers argue that this pattern is paradoxical because interacting congruency effects suggest domain-general control (i.e., conflict resolution processes that operate in multiple domains of distractor processing) while additive CSEs suggest domain-specific control (i.e., conflict resolution processes that operate in only a single domain of distractor processing) (Schlaghecken & Maylor, 2020). Others argue that this pattern makes it impossible to distinguish at all between domain-specific and domain-general CSEs (Egner, 2008). The logic here is that one can only determine whether control processes underlying the CSE operate in a domain-specific or domain-general manner when there are at least two independent domains of distractor processing. According to additive factors logic (Sternberg, 1969), additive congruency effects indicate two independent domains (i.e., processing stages) of distractor processing. In contrast, interacting congruency effects indicate a common, single domain (i.e., processing stage) of distractor processing (Egner, 2008; Kornblum, 1994). Critically, if there is only one domain of distractor processing, there is no “domain boundary” across which control processes can produce a domain-general CSE. Thus, in this situation, it is logically impossible to distinguish between domain-specific and domain-general CSEs.

### The reactivation aversion effect (RAE)

Schlaghecken et al. (2011) proposed an intriguing explanation for the puzzling data pattern described above. Here, an interaction between congruency effects within trials (henceforth, a “within-trial interaction”), reflects meta-cognitive confusion. That is, it reflects a confound in the experimental design, rather than (a) domain-general control within trials (Schlaghecken & Maylor, 2020) or (b) that two distractors are processed in the same domain (Egner, 2008). Critically, this account predicts that a more straightforward pattern of additive congruency effects and additive CSEs should appear when researchers control for this confound.

Consider Schlaghecken & Maylor’s (2020) hybrid prime-Simon task. Here, an initial prime distractor (e.g., a left or right arrow) is followed by a target (e.g., another left or right arrow) whose location in the display (e.g., left or right) serves as the Simon distractor. Schlaghecken & Maylor suggest that a within-trial interaction appears in this task because a *reactivation aversion effect* (RAE) delays responses in prime-compatible/Simon-incongruent trials^1^. Here, the initial prime distractor (e.g., a left arrow) activates the response that will ultimately be required by the upcoming target (e.g., left). The subsequent Simon distractor, however, activates the opposite response (e.g., right), which leads the cognitive system to “abandon” the response activated by the prime distractor. Thus, responding to the target requires reactivating a recently abandoned response (e.g., left). Schlaghecken & Maylor argued that this requirement leads to “meta-cognitive confusion”, which slows performance.

It is important to emphasize that meta-cognitive confusion comes from reactivating a previously abandoned response, rather than from two distractors cueing different responses. As an example of the latter, consider prime-incompatible/Simon-congruent trials. In these trials, the initial prime distractor (e.g., a left arrow) activates a response (e.g., left) that is opposite to the response the upcoming target will require (e.g., right). The Simon distractor then activates the upcoming target response (e.g., right). Critically, when the target (e.g., a right arrow) appears, the cognitive system can make the correct response (e.g., right) without reactivating the response that was activated by the initial prime distractor (e.g., left). Consequently, in this trial type, there is no meta-cognitive confusion due to reactivating a previously abandoned prime response.

**Fig. 1:**
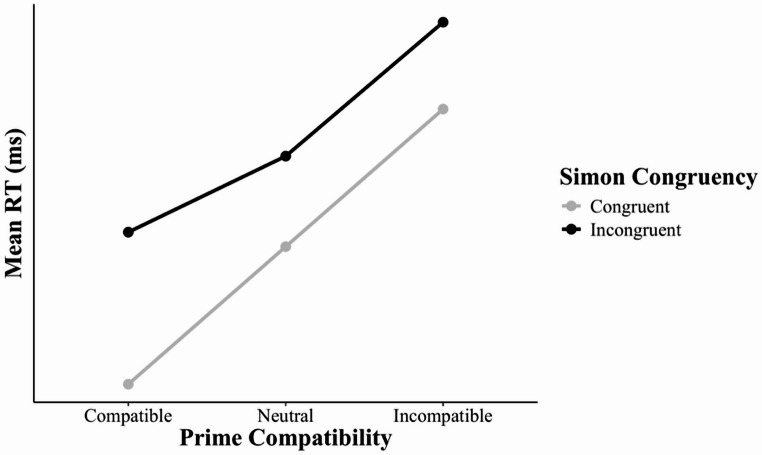
Hypothetical example of a within-trial interaction driven solely by the RAE. Selective slowing of responses in prime-compatible/Simon-incongruent trials produces a larger Simon effect in prime-compatible trials than in either prime-neutral or prime-incompatible trials. In contrast, the Simon effect does not differ between prime-neutral trials and prime-incompatible trials. RAE = reactivation aversion effect

To test the RAE account of the within-trial interaction, Schlaghecken and Maylor (2020) included prime-neutral trials in a hybrid prime-Simon task. Here, the prime was a plus sign that was not mapped to a task-relevant response. The authors asserted that an interaction between prime compatibility (*compatible*,* neutral*) and Simon congruency (congruent, incongruent), which *includes* prime-compatible/Simon-incongruent trials, would indicate an influence of the RAE on the within-trial interaction. They also asserted that an interaction between prime compatibility (*neutral*,* incompatible*) and Simon congruency (congruent, incongruent), which *excludes* prime-compatible/Simon-incongruent trials, would indicate an influence of domain-general mechanisms for processing concurrent distractions on the within-trial interaction. As a hypothetical example, Fig. 1 illustrates a within-trial interaction driven solely by the RAE (i.e., by a selective slowing of responses in prime-compatible/Simon-incongruent trials).

Critically, unlike in some prior studies (e.g., Rey-Mermet, 2020), Schlaghecken and Maylor (2020) controlled for contingency learning confounds (e.g., Schmidt & De Houwer, 2011), which might lead to an interaction between congruency effects via mechanisms unrelated to domain-specific or domain-general control. Such confounds are often present in tasks with more than two possible stimuli and responses. The reason is that researchers typically present each unique congruent distractor-target pair more frequently than each unique incongruent distractor-target pair to ensure that each trial type (i.e., congruent and incongruent) appears 50% of the time as in a two-choice task. Under such conditions, each distractor becomes more strongly associated with the congruent response than with each possible incongruent response. These asymmetric associations increase the size of the congruency effect. They also make it difficult for researchers to determine whether interacting congruency effects index interacting (a) control processes or (b) contingency learning processes.

Schlaghecken and Maylor’s (2020) findings suggested that the RAE can explain the within-trial interaction in the hybrid prime-Simon task and thereby explain the generality-specificity paradox. First, consistent with an influence of the RAE, there was an interaction between prime compatibility (*compatible*,* neutral*) and Simon congruency (congruent, incongruent). Second, consistent with an influence of domain-specific, rather than domain-general, mechanisms, there were both (1) additive effects of prime compatibility (*neutral*,* incompatible*) and Simon congruency (congruent, incongruent) when controlling for the RAE and (2) additive CSEs related to the two distractors^2^. Given these findings, the authors concluded that there are domain-specific mechanisms for (a) processing concurrent distractions caused by each of two irrelevant stimuli and (b) minimizing future distractions caused by the same stimuli. More broadly, they suggested that the RAE may explain the puzzling pattern of interacting congruency effects and additive CSEs in hybrid distractor-interference tasks.

### Competing accounts of the within-trial interaction

Although the RAE account explains the within-trial interaction in Schlaghecken & Maylor’s (2020) study, there are competing accounts of this interaction. These accounts suggest that other mechanisms may also contribute to the within-trial interaction. Consequently, they suggest that the RAE may not always explain the puzzling pattern of interacting congruency effects and additive CSEs that researchers report frequently in hybrid distractor-interference tasks. Given the importance of understanding this puzzling pattern for distinguishing between domain-specific and domain-general control, we next review two competing accounts of the within-trial interaction. To our knowledge, neither account has been investigated while simultaneously controlling for both the RAE and contingency learning confounds.

### The conflict type account

Kornblum and colleagues (1994) proposed a *conflict type account* of the within-trial interaction. In this account, interactions between congruency effects depend on whether two distractors trigger the same or different types of conflict in Kornblum and colleagues’ (1990) taxonomy (see Egner, 2008 for a review). This taxonomy describes several types of conflict. For example, stimulus-stimulus (S-S) conflict occurs in incongruent trials of the flanker task because perceptual and/or semantic features of the distractors conflict with those of the target. As another example, stimulus-response (S-R) conflict occurs in incongruent trials of the Simon task because the target’s location on the screen conflicts with the location of the correct response on the keyboard.

According to the conflict type account, additive congruency effects occur when each distractor triggers a different type of conflict. For example, additivity occurs when one distractor triggers S-S conflict while another distractor triggers S-R conflict (e.g., in a hybrid Stroop-Simon task) (e.g., Kornblum, 1994). In contrast, interacting congruency effects occur when two distractors trigger the same type of conflict (e.g., when both trigger S-S conflict in a hybrid Stroop-flanker task). Consistent with this account, Kornblum’s initial findings revealed that congruency effects stemming from S-S and S-R conflicts sum additively (Kornblum, 1994)^3^. Subsequent findings, however, challenged the conflict type account by revealing a within-trial interaction even when two distractors trigger different types of conflict (e.g., Stoffels & van der Mole 1988; Hommel, 1997; Wendt et al., 2006; Treccani et al., 2009; Fruhholz et al. 2011; Rey-Mermet & Gade, 2016; Weissman, 2020; Rey-Mermet, 2020; Rey-Mermet et al., 2021). None of these studies, however, controlled for the RAE.

### The operating space account

Recently, Rey-Mermet (2020) proposed that a within-trial interaction is possible only when at least one of the two congruency effects is relatively large. This proposal fits with the view that this interaction requires “operating space” (Sanders, 1980; Hommel, 1997). In the hybrid Stroop-flanker task, for example, it may be more difficult to observe a smaller Stroop effect in flanker-incongruent trials than in flanker-congruent trials if the Stroop effect is already quite small in flanker-congruent trials. Consistent with this *operating space account*, Rey-Mermet (2020) reported within-trial interactions between the Stroop and flanker effects when the Stroop effect was large but not when it was small. To explain this outcome, Rey-Mermet (2020) suggested that small congruency effects indicate that a distraction is not processed to a degree that enables it to interact with other processes (e.g., the processing of another distractor).

What causes a within-trial interaction when there is sufficient operating space (i.e., a large congruency effect)? Rey-Mermet and colleagues suggest that such interactions stem from overlapping response selection and/or execution processes when two distractors prime the same response (Rey-Mermet & Gade, 2016; Rey-Mermet et al., 2019, 2021). For example, when both distractors are congruent with the target, the activation of the congruent response induced by one distractor (e.g., flanker) may allow the other distractor (e.g., Simon) to further activate that response in proportion to its (already high) level of activation (Rey-Mermet et al., 2021). Rey-Mermet et al. (2021) suggest that such “multiplicative priming” of the congruent (i.e., correct) response may speed performance, leading to a within-trial interaction. Consistent with this view, these authors reported especially fast responses in the flanker-congruent/Simon-congruent trials of a hybrid flanker-Simon task. Furthermore, as we described earlier, a within-trial interaction did not appear in Schlaghecken & Maylor’s (2020) hybrid prime-Simon task when prime-compatible/Simon-congruent trials were excluded from the analyses by excluding all prime-compatible trials. Multiplicative priming of the incongruent response in prime-incompatible/Simon-incongruent trials might also speed performance by allowing the cognitive system to more rapidly categorize the incongruent response as incorrect and reject that response after the target appears (Treccani et al., 2009, 2012). Of course, processes other than those related to response selection and/or response execution (e.g., overlapping conflict resolution processes) may also – or instead – lead to a within-trial interaction when there is sufficient operating space.

### The present study

Schlaghecken and Maylor (2020) offer a rigorous (i.e., “RAE confound-minimized”) approach for investigating the within-trial interaction in hybrid distractor-interference tasks. It is unclear, however, whether the RAE is the only cognitive mechanism that contributes to this interaction after controlling for contingency learning. Since understanding this interaction is important for drawing conclusions about whether cognitive control operates in a domain-specific or domain-general manner (Egner, 2008; Schlaghecken & Maylor, 2020), the present goal is to (1) investigate whether this interaction can occur even while controlling for both contingency learning and the RAE and, if it can, (2) gain insights into whether the conflict type account or the operating space account provides a better explanation of when this interaction occurs.

To this end, we vary across three RAE- and contingency-learning-controlled experiments both (1) whether two distractors engender the same or different types of conflict with the target and (2) the size of the prime compatibility effect. If the RAE is the only mechanism that produces a within-trial interaction after controlling for contingency learning, as Schlaghecken & Maylor’s (2020) results suggest, then we should not observe such an interaction. If the RAE is not the only mechanism that produces such an interaction, however, then the conflict type and operating space accounts make distinct predictions about when a “RAE-controlled” within-trial interaction is more likely to appear. The conflict type account predicts that such an interaction is more likely when two distractors engender the same type of conflict (Experiments 1 and 2) relative to different types of conflict (Experiment 3) (Kornblum, 1994). In contrast, the operating space account predicts that such an interaction is more likely when the prime compatibility effect is relatively large (Experiments 2 and 3) as compared to relatively small (Experiment 1) (Rey-Mermet, 2020).

### Experiment 1

In Experiment 1, we employ a hybrid prime-flanker task. In each trial, participants indicate whether a central target square is red or green. There are two distractors, each of which engenders S-S conflict with the target. The first distractor is a prime word (“Red”, “Green”, or “Cross”) that should produce a relatively small prime compatibility effect because it does not possess target-defining features that capture attention (i.e., the color “red” or the color “green”) (Buetti et al., 2014). As in Schlaghecken and Maylor (2020), we include a neutral prime distractor condition, wherein the prime is the word “Cross”, to control for the effect of the RAE when assessing the within-trial interaction. The second distractor is composed of four flanker squares – two on each side of the central target – that are all colored red or all colored green.

The three accounts under investigation make the following predictions. The RAE account does not predict a RAE-controlled within-trial interaction. In contrast, the conflict type account predicts that such an interaction is likely because the two distractors engender the same type of conflict. Finally, the operating space account predicts that such an interaction is unlikely if the prime compatibility and flanker congruency effects are both relatively small as we expect.

## Methods

### Power analyses

We powered Experiment 1 to observe two effects. The first effect is the portion of the within-trial interaction that is influenced by the RAE (henceforth, the “RAE”), which is the interaction between prime compatibility (*compatible*,* neutral*) and flanker congruency (congruent, incongruent). The second effect is the portion of the within-trial interaction that is not influenced by the RAE (henceforth, the “RAE-controlled within-trial interaction”), which is the interaction between prime compatibility (*neutral*,* incompatible*) and flanker congruency (congruent, incongruent).

We determined that a sample size of 56 participants would be sufficient to observe these interactions for two reasons. First, this sample size matched that of Schlaghecken and Maylor (2020) (*N* = 50) who assessed the same interactions and observed a RAE in a similar task. Second, power analyses conducted with G*power 3.1.9.7 revealed that 56 participants would provide over 95% power (with an alpha of 0.05) to observe an effect associated with a partial eta squared value of 0.20. Since this value is smaller than the smallest partial eta squared value for the RAE in Schlaghecken and Maylor (2020), we reasoned that it would be sufficient to detect the RAE. We also judged that it would provide a reasonable starting point for investigating a within-trial interaction between prime compatibility (*neutral*,* incompatible*) and Simon congruency (congruent, incongruent) that is not confounded with contingency learning or the RAE^4^.

### Participants

Sixty-four undergraduate students from the University of Michigan participated in Experiment 1 for course credit. We excluded five participants who performed with less than 75% accuracy and three participants who reported abnormal vision. Thus, the analyses we present in Experiment 1 include the remaining 56 participants (16 males, 40 females; mean age, 18.9; age range 18–22), each of whom reported normal or corrected-to-normal vision and hearing.

### Stimuli and apparatus

We presented stimuli and recorded responses with PsychoPy 2022.2.4 (Peirce et al., 2019) running on a Windows 10 PC. All stimuli appeared at the center of the screen against a black background. At the beginning of each block, a white fixation cross (~ 0.63° x ~ 0.63°) appeared for 1.8 s. Following an intervening 200 ms blank screen, the trials began.

**Fig. 2:**
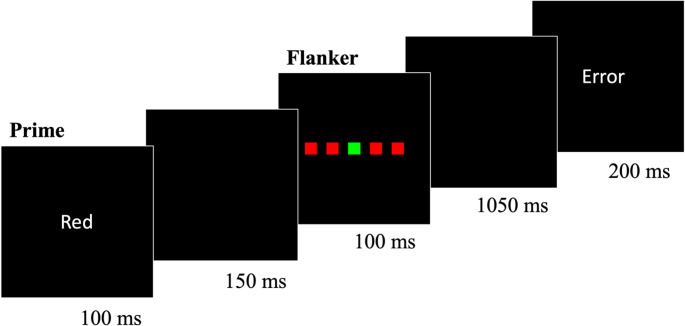
Example of a prime-flanker task trial from Experiment 1. Note. Each 1.6-s trial consisted of a prime word distractor (“Red”, “Green”, or “Cross”) followed by a flanker array. Participants were instructed to make a key press to identify the color (*red* or *green*) of the central square in the flanker array (i.e., the target) as quickly and accurately as possible. The duration of each trial component appears beneath its respective frame

Each trial (see Fig. 2,^5^) consisted of a prime distractor (duration, 100 ms), a blank screen (duration, 150 ms), a flanker array (duration, 100 ms), a second blank screen (duration, 1050 ms), and, in some trials (see below), a feedback message (duration, 200 ms). The prime distractor was the word “Red” (~ 3.96° x ~ 1.87°), “Green” (~ 6.87° x ~ 1.87°), or “Cross” (~ 5.93° x ~ 1.87°) printed in white. The flanker array (6.04° x ~ 0.94°) consisted of five squares (~ 0.94° x ~ 0.94°) spaced equally apart on the x-axis (~ 0.26°). Each square was red or green. In any given trial, the four outer squares (i.e., the flanker distractor) all appeared in a single color (red or green). The central square (i.e., the target) appeared in the same color as the flankers or in a different color.

### Task

We instructed participants to identify the color of the central target in the flanker array as quickly and as accurately as possible. On a QWERTY keyboard, half the participants pressed “z” (left index finger) or “m” (right index finger) for *green* and *red*, respectively. The other half of participants used the reverse stimulus-response (S-R) mapping. We randomized these two S-R mappings across participants. More specifically, participants with odd participant numbers had one S-R mapping while participants with even participant numbers had the other S-R mapping.

We allowed participants to respond anytime up to 1000 ms following the onset of the flanker array. If a participant responded before the flanker array appeared, pressed the wrong key after the flanker array appeared, or responded more than 1000 ms after the flanker array appeared, then the word “Error” (~ 2.92° x ~ 0.94°) appeared centrally in white for 200 ms. The error message appeared from 1150 ms to 1350 ms after the flanker array appeared. If a participant pressed the correct key after the flanker array appeared but before the response deadline (i.e., 1000 ms), then a 200 ms blank screen appeared instead of the error message.

### Experimental design

As we described above, each trial included a prime distractor (i.e., “Red”, “Green”, or “Cross”), a flanker distractor (i.e., four red squares or four green squares) and a target (i.e., a red square or a green square). There were two distinct sets of distractor-target relationships. First, each trial was prime-compatible, prime-neutral, or prime-incompatible. In prime-compatible trials, the prime word cued the same response as the target color (i.e., RED-*red* or GREEN-*green*). In prime-neutral trials, the prime did not cue a task-relevant response (i.e., CROSS-*red* or CROSS-*green*). In prime-incompatible trials, the prime word cued a different response than the target color (i.e., RED-*green* or GREEN-*red*). Second, each trial was flanker-congruent or flanker-incongruent. In flanker-congruent trials, the flankers’ color and the target’s color cued the same response (i.e., *red*-*red* or *green*-*green*). In flanker-incongruent trials, however, the flankers’ color and the target’s color cued different responses (i.e., *red*-*green* or *green*-*red*).

We employed a fully crossed, factorial design. Consequently, in every block, each of the two possible combinations of flanker congruency (congruent, incongruent) appeared equally often in each of the three possible combinations of prime compatibility (compatible, neutral, incompatible). Since there were 108 trials in each test block, there were 18 trials in each of the six possible combinations of prime compatibility and flanker congruency described above. Over the course of the experiment, participants completed ten test blocks (1080 test trials in total).

Each 108-trial test block consisted of nine sequences of 12 randomly ordered trials. Each trial represented one of the 12 possible combinations of three prime distractor words, two flanker distractor colors, and two target colors. By presenting each of the 12 possible combinations equally often, we avoided contingency learning confounds. The program generated a new 12-trial sequence nine times in each block to create 108 trials in total. Consequently, the 108 trials in each block appeared in a different, pseudo-randomized order, separately for every participant.

### Procedure

Participants completed the experiment in private testing rooms. Before beginning the experiment, the researcher positioned the participant in a chinrest, ensuring that the participant’s eyes were approximately 55 cm from the center of the computer screen. After positioning the participant, the researcher administered informed consent forms and verbally reviewed written task instructions on the computer screen before beginning the task.

Each participant first completed a single practice block of 48 trials that was divided into four sub-blocks of 12 trials. A reminder of the task instructions appeared before each practice sub-block and before each of the following test blocks. The researcher stayed in the room with the participant to monitor their performance during the practice trials. To ensure that each participant understood the task and could perform it successfully, the researcher implemented a second practice block if it appeared to the researcher that a participant performed with low accuracy (e.g., 70–75% or lower) during the initial 48-trial practice block. The researcher left the room after the participant completed the practice block. The participant then completed the ten test blocks. Following the test blocks, the participant viewed a debriefing on the computer screen that described the purpose of the experiment. The participant was also allowed to ask the researchers any questions they had about the study.

### Data analyses

We excluded all practice trials from the analyses. Overall, participants performed with high accuracy on the remaining test trials as only 8.7% of these trials were errors. As we explain next, we also excluded some of the test trials on a participant-by-participant basis, separately for the analyses of mean RT and mean ER.

In the analysis of mean RT, we excluded trials with an error or omitted response^6^ and trials that followed a trial with an error or omitted response. We excluded trials after errors to avoid the possibility that post-error slowing (e.g., Rabbit, 1966) might influence our measurements of within-trial interactions. We also excluded outliers (5.3% of the remaining trials). Outliers were trials with RTs greater than 3*S_n_ (Rousseeuw & Croux, 1993), calculated separately for each of the six combinations of prime compatibility (compatible, neutral, incompatible) and flanker congruency (congruent, incongruent). S_n_ is the median absolute distance of each data point in a condition from every other data point in the same condition. We called a trial an “outlier” if the median absolute distance of its RT from every other RT in the same condition was more than three times its condition-specific S_n_ value. Using S_n_ cutoffs to identify outliers is preferable to using standard deviation cutoffs in the present study. Indeed, unlike the calculation of standard deviation, the calculation of S_n_ assumes neither a measure of central tendency (e.g., a mean) nor a normal distribution (Jones, 2019). The lack of such assumptions is desirable given that RT distributions are skewed.

In the analysis of mean error rate (ER), we excluded the same trials as in the mean RT analysis except for trials that contained an error, as these trials formed the dependent measure. Since we used only correct RTs to identify outliers in the analysis of mean RT but used both correct and error RTs to identify outliers in the analysis of mean ER, the percentage of outliers in the analysis of mean ER differed slightly from that in the analysis of mean RT. More specifically, 5.4% of the trials were outliers in the analysis of mean ER. Tables 1 and 2 present mean RT and mean ER in each condition, respectively, following these exclusions. Table 3 presents the number of trials in each condition following trial exclusions separately for mean RT and mean ER.

**Table 1:** Mean RTs, standard errors, and flanker effects (in milliseconds) in experiment 1. The flanker effects we report in the table were calculated after rounding the group-averaged mean RTs for each condition. Therefore, these values may differ (e.g., by 1 ms) from the flanker effects that we report in the text, which we calculated before rounding the mean RTs for each condition. RT = response time; cC = prime-compatible, flanker-congruent; cI = prime-compatible, flanker-incongruent; nC = prime-neutral, flanker-congruent; nI = prime-neutral, flanker-incongruent; iC = prime-incompatible, flanker-congruent; iI = prime-incompatible, flanker-incongruent; flanker effect = incongruent–congruent. Std. Error = standard error of the mean across participants

Prime Compatibility	Flanker Congruency	Mean RT	Std. Error	Flanker Effect
Compatible	Congruent (cC)	439	5	42
	Incongruent (cI)	481	6	
Neutral	Congruent (nC)	443	5	35
	Incongruent (nI)	478	6	
Incompatible	Congruent (iC)	466	5	32
	Incongruent (iI)	498	7

**Table 2:** Mean ERs, standard errors, and flanker effects (in percent) in Experiment 1. ER = error rate; cC = prime-compatible, flanker-congruent; cI = prime-compatible, flanker-incongruent; nC = prime-neutral, flanker-congruent; nI = prime-neutral, flanker-incongruent; iC = prime-incompatible, flanker-congruent; iI = prime-incompatible, flanker-incongruent; flanker effect = incongruent–congruent. Std. Error = standard error of the mean across participants

Prime Compatibility	Flanker Congruency	Mean ER	Std. Error	Flanker Effect
Compatible	Congruent (cC)	3.5%	0.4%	4.1%
	Incongruent (cI)	7.6%	0.6%	
Neutral	Congruent (nC)	3.8%	0.4%	4.0%
	Incongruent (nI)	7.8%	0.6%	
Incompatible	Congruent (iC)	5.9%	0.6%	4.3%
	Incongruent (iI)	10.2%	0.9%

**Table 3:** Mean number of trials per condition following exclusions in Experiment 1. RT = reaction time; ER = error rate; cC = prime-compatible, flanker-congruent; cI = prime-compatible, flanker-incongruent; nC = prime-neutral, flanker-congruent; nI = prime-neutral, flanker-incongruent; iC = prime-incompatible, flanker-congruent; iI = prime-incompatible, flanker-incongruent

Prime Compatibility	Flanker Congruency	RT Analysis	ER Analysis
Compatible	Congruent (cC)	147	152
	Incongruent (cI)	143	155
Neutral	Congruent (nC)	146	152
	Incongruent (nI)	142	154
Incompatible	Congruent (iC)	141	150
	Incongruent (iI)	137	153

After excluding the trials described above, we analyzed the data with a series of repeated-measures MANOVAs using IBM SPSS Statistics for Macintosh (Version 29.0). We chose to employ MANOVAs because they do not make assumptions about sphericity that are often violated when a factor has more than two levels as is the case in the present experiments. We conducted these MANOVAs separately for mean RT and mean ER using Pillai’s trace to assess significance. Mean RT was our primary dependent measure. However, we also analyzed mean ER to assess potential speed-accuracy tradeoffs.

We first conducted an omnibus 3 × 2 repeated-measures MANOVA. The factors were prime compatibility (compatible, neutral, incompatible) and flanker congruency (congruent, incongruent). If the interaction was significant, we conducted three follow-up 2 × 2 MANOVAS^7^ that included different levels of prime compatibility. The first MANOVA assessed the within-trial interaction without controlling for the RAE as Egner (2008) describes; that is, this MANOVA assessed the interaction between prime compatibility (*compatible*,* incompatible*) and flanker congruency (congruent, incongruent). The second MANOVA assessed the contribution of the RAE to the within-trial interaction; that is, the interaction between prime compatibility (*compatible*,* neutral*) and flanker congruency (congruent, incongruent) as Schlaghecken and Maylor (2020) describe. The third MANOVA assessed the “RAE-controlled” within-trial interaction; that is, the interaction between prime compatibility (*neutral*,* incompatible*) and flanker congruency (congruent, incongruent) (Schlaghecken & Maylor, 2020).

### Transparency and openness

In our preregistration for Experiment 1, we provide the rationale for the manipulations, dependent measures, sample size, and data exclusions. Our preregistration, data analysis scripts, raw data, and task scripts are freely available on the Open Science Framework (https://osf.io/ux8e2/).

## Results

### Mean RT

**Fig. 3:**
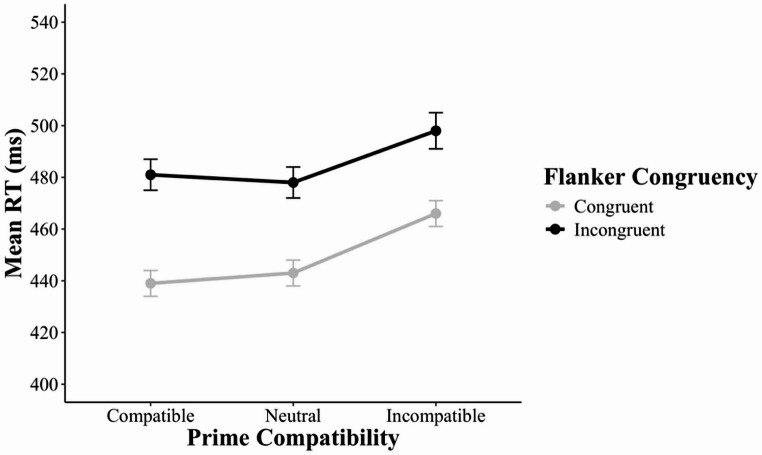
Mean reaction time as a function of prime compatibility and flanker congruency in Experiment 1. Error bars indicate ±1 standard error of the condition mean across participants. RT = reaction time

The omnibus 3 × 2 MANOVA revealed three significant effects. First, there was a main effect of prime compatibility, *F*(2, 54) = 99.48, *p* < 0.001, *η*_*p*_^*2*^ = 0.79, because mean RT varied among prime-compatible (460 ms), prime-neutral (460 ms), and prime-incompatible (482 ms) trials. Second, there was a main effect of flanker congruency, *F*(1, 55) = 270.67, *p* < 0.001, *η*_*p*_^*2*^ = 0.83, because mean RT was longer in flanker-incongruent trials (486 ms) than in flanker-congruent trials (449 ms). Third, there was an interaction between prime compatibility and flanker congruency, *F*(2, 54) = 14.42, *p* < 0.001, *η*_*p*_^*2*^ = 0.35 (Fig. 3).

Given the significant interaction in the omnibus MANOVA, we continued with more focused 2 × 2 MANOVAs. The first MANOVA revealed a within-trial interaction without controlling for the RAE – that is, an interaction between prime compatibility (*compatible*,* incompatible*) and flanker congruency (congruent, incongruent), *F*(1, 55) = 29.36, *p* < 0.001, *η*_*p*_^*2*^ = 0.35 – because the flanker effect was larger in prime-compatible trials (43 ms) relative to prime-incompatible trials (32 ms). The second MANOVA revealed a RAE – that is, an interaction between prime compatibility (*compatible*,* neutral*) and flanker congruency (congruent, incongruent), *F*(1, 55) = 10.53, *p* = 0.002, *η*_*p*_^*2*^ = 0.16 – because the flanker effect was larger in prime-compatible trials (43 ms) than in prime-neutral trials (35 ms). The third MANOVA did not reveal a significant RAE-controlled within-trial interaction – that is, an interaction between prime compatibility (*neutral*,* incompatible*) and flanker congruency (congruent, incongruent), *F*(1, 55) = 3.27, *p* = 0.076, *η*_*p*_^*2*^ = 0.06 – because the flanker effect did not differ between prime-neutral trials (35 ms) and prime-incompatible trials (32 ms).

### Mean ER

The omnibus 3 × 2 MANOVA revealed two significant effects. First, there was a main effect of prime compatibility, *F*(2, 54) = 11.27, *p* < 0.001, *η*_*p*_^*2*^ = 0.29, as mean ER varied among prime-compatible (5.5%), prime-neutral (5.8%), and prime-incompatible (8.0%) trials. Second, there was a main effect of flanker congruency, *F*(1, 55) = 108.74, *p* < 0.001, *η*_*p*_^*2*^ = 0.66, as mean ER was higher in flanker-incongruent trials (8.6%) than in flanker-congruent trials (4.4%). The two-way interaction between prime compatibility (compatible, neutral, incompatible) and flanker congruency (congruent, incongruent) was not significant, *F*(2, 54) < 1. Therefore, we did not conduct additional, more focused 2 × 2 MANOVAs.

## Discussion

Consistent with the RAE account, we did not observe a significant RAE-controlled within-trial interaction. This finding is also consistent with the operating space account, assuming that neither the prime compatibility effect nor the flanker congruency effect was large enough to allow for a within-trial interaction (Rey-Mermet, 2020). In contrast, this finding is inconsistent with the conflict type account, in which a within-trial interaction should appear when two distractors engender the same type of conflict (Kornblum, 1994) as they did in Experiment 1.

A potential limitation of Experiment 1 is that the RAE-controlled within-trial interaction approached significance (*p* = ~ 0.08). Thus, one might argue that the results are not convincingly more consistent with the RAE and operating space accounts than with the conflict type account. For this reason, we refrain from making strong conclusions before reviewing the findings from subsequent experiments.

### Experiment 2

In Experiment 2, we investigated whether a RAE-controlled within-trial interaction appears when the prime compatibility effect is relatively large (cf., Rey-Mermet, 2020), which provides more operating space for an interaction to occur (Hommel, 1997). To accomplish this goal, we replaced the prime words “Red”, “Green”, and “Cross” with red, green, and white squares, respectively. We reasoned that this change would increase the size of the prime compatibility effect by making the congruent and incongruent prime distractors identical to potential targets. Consistent with our reasoning, prior findings indicate that distractors that possess target-defining perceptual features (e.g., target-defining colors) capture attention (e.g., Serences et al., 2005; Moore & Weissman, 2010), which should lead to larger compatibility effects (e.g., Buetti et al., 2014).

While the RAE account does not predict a RAE-controlled within-trial interaction, the conflict type and operating space accounts both predict that such an interaction is likely in the present experiment. As in Experiment 1, the conflict type account makes this prediction because each distractor triggers the same type of conflict with the target (i.e., S-S conflict). The operating space account also makes this prediction if the prime compatibility effect is much larger than in Experiment 1 as we expect. Moreover, the operating space account, which posits a role for the size of the prime compatibility effect in producing the within-trial interaction, could more easily explain why a RAE-controlled within-trial interaction appears when the prime compatibility effect is large (i.e., as we expect in Experiment 2) but not small (i.e., in Experiment 1).

### Methods

#### Power analyses

As in Experiment 1, we powered Experiment 2 to observe both (1) a RAE – i.e., an interaction between prime compatibility (*compatible*,* neutral*) and flanker congruency (congruent, incongruent) – and (2) a within-trial interaction that is not influenced by the RAE – i.e., an interaction between prime compatibility (*neutral*,* incompatible*) and flanker congruency (congruent, incongruent). Employing the same logic as in Experiment 1, we determined with G*Power 3.1.9.7 that 56 participants would be sufficient to observe these interactions. Although Experiment 1 did not yield the latter interaction, increasing the visual similarity between the set of prime distractors and the set of targets should make the prime compatibility effect larger than in Experiment 1. Therefore, a larger RAE-controlled within-trial interaction than in Experiment 1 might appear if “operating space” influences the magnitude of the within-trial interaction.

#### Participants

Sixty-three undergraduate students from the University of Michigan participated in Experiment 2 for course credit. None had participated in Experiment 1. We excluded the data from seven participants: six who performed with less than 75% accuracy and one who reported abnormal hearing. Consequently, the analyses that we present for Experiment 2 include the remaining 56 participants (15 males, 41 females; mean age, 19.0; age range 18–21), each of whom reported normal or corrected-to-normal vision and hearing.

#### Stimuli and apparatus

**Fig. 4:**
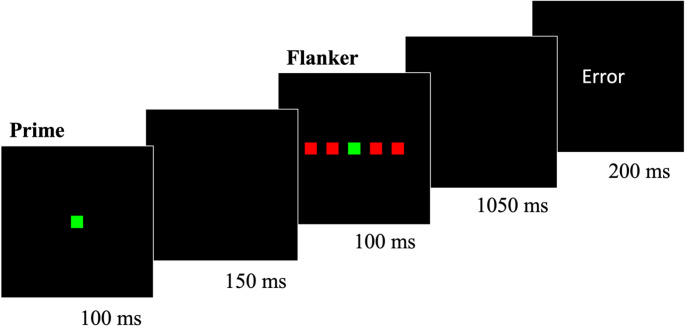
Example of a prime-flanker task trial from Experiment 2. Note. Experiment 2 was identical to Experiment 1 with the exception that the prime distractor was a colored (i.e., red, green, or white) square. The white prime distractor appeared only in prime-neutral trials

The stimuli and apparatus were identical to those in Experiment 1 with one exception. The prime distractor in each trial was a red, green, or white square (~ 0.94° x ~ 0.94°; see Fig. 4), rather than a color word as in Experiment 1. Consequently, both the prime distractor and the flanker distractors were colored squares in Experiment 2. Analogous to the word “Cross” in Experiment 1, the white (i.e., neutral) prime square was not mapped to a task-relevant response.

#### Task and experimental design

The task and experimental design were identical to those in Experiment 1.

### Procedure

The procedure was identical to that in Experiment 1.

### Data analyses

The data analyses were identical to Experiment 1. On average, 10.2% of all trials were errors. In the analysis of mean RT, 5.4% of the trials were outliers. In the analysis of mean ER, 5.8% of the trials were outliers. Tables 4 and 5 show each condition’s mean RT and mean ER, respectively. Table 6 presents the number of trials in each condition following trial exclusions separately for mean RT and mean ER.

**Table 4:** Mean RTs, standard errors, and flanker effects (in milliseconds) in Experiment 2. The flanker effects we report in the table were calculated after rounding the group-averaged mean RTs for each condition. Therefore, these values may differ (e.g., by 1 ms) from the flanker effects that we report in the text, which we calculated before rounding the mean RTs for each condition. RT = response time; cC = prime-compatible, flanker-congruent; cI = prime-compatible, flanker-incongruent; nC = prime-neutral, flanker-congruent; nI = prime-neutral, flanker-incongruent; iC = prime-incompatible, flanker-congruent; iI = prime-incompatible, flanker-incongruent; flanker effect = incongruent–congruent. Std. Error = standard error of the mean across participants

Prime Compatibility	Flanker Congruency	Mean RT	Std. Error	Flanker Effect
Compatible	Congruent (cC)	443	6	57
	Incongruent (cI)	500	7	
Neutral	Congruent (nC)	476	6	29
	Incongruent (nI)	505	7	
Incompatible	Congruent (iC)	530	9	−3
	Incongruent (iI)	527	8

**Table 5:** Mean ERs, standard errors, and flanker effects (in percent) in Experiment 2. Note. ER = error rate; cC = prime-compatible, flanker-congruent; cI = prime-compatible, flanker-incongruent; nC = prime-neutral, flanker-congruent; nI = prime-neutral, flanker-incongruent; iC = prime-incompatible, flanker-congruent; iI = prime-incompatible, flanker-incongruent; flanker effect = incongruent–congruent. Std. Error = standard error of the mean across participants

Prime Compatibility	Flanker Congruency	Mean ER	Std. Error	Flanker Effect
Compatible	Congruent (cC)	2.8%	0.3%	4.1%
	Incongruent (cI)	6.9%	0.7%	
Neutral	Congruent (nC)	4.1%	0.4%	1.4%
	Incongruent (nI)	5.5%	0.5%	
Incompatible	Congruent (iC)	12.2%	1.0%	−2.7%
	Incongruent (iI)	9.5%	0.8%

**Table 6:** Mean number of trials per condition following exclusions in Experiment 2. RT = reaction time; ER = error rate; cC = prime-compatible, flanker-congruent; cI = prime-compatible, flanker-incongruent; nC = prime-neutral, flanker-congruent; nI = prime-neutral, flanker-incongruent; iC = prime-incompatible, flanker-congruent; iI = prime-incompatible, flanker-incongruent

Prime Compatibility	Flanker Congruency	RT Analysis	ER Analysis
Compatible	Congruent (cC)	144	148
	Incongruent (cI)	140	150
Neutral	Congruent (nC)	141	147
	Incongruent (nI)	142	150
Incompatible	Congruent (iC)	130	149
	Incongruent (iI)	134	149

### Transparency and openness

In our preregistration for Experiment 2, we provide the rationale for the manipulations, dependent measures, sample size, and data exclusions. Our preregistration, data analysis scripts, raw data, and task scripts are freely available on the Open Science Framework (https://osf.io/dz6qj/).

## Results

### Mean RT

**Fig. 5:**
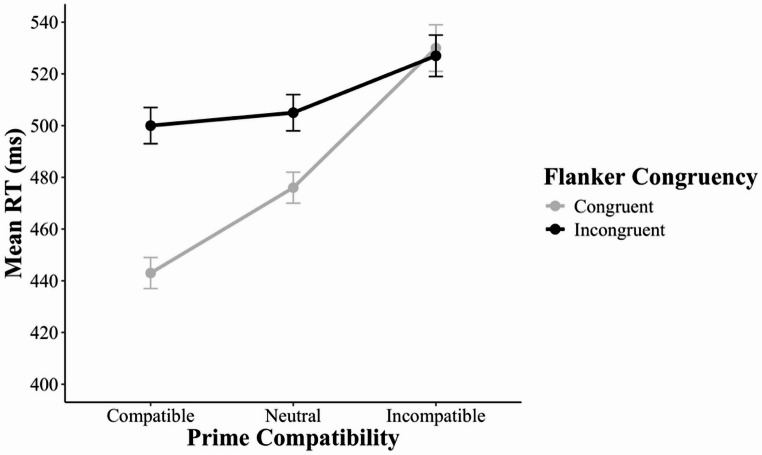
Mean reaction time (in milliseconds) as a function of prime compatibility and flanker congruency in Experiment 2. Error bars indicate ± 1 standard error of the condition mean across participants. RT = reaction time

The omnibus 3 × 2 MANOVA revealed three significant effects. First, there was a main effect of prime compatibility, *F*(2, 54) = 103.55, *p* < 0.001, *η*_*p*_^*2*^ = 0.79, as mean RT varied among prime-compatible (471 ms), prime-neutral (491 ms), and prime-incompatible (528 ms) trials. Second, there was a main effect of flanker congruency, *F*(1, 55) = 254.46, *p* < 0.001, *η*_*p*_^*2*^ = 0.82, as mean RT was longer in flanker-incongruent trials (510 ms) than in flanker-congruent trials (483 ms). Third, there was a two-way interaction between prime compatibility (compatible, neutral, incompatible) and flanker congruency (congruent, incongruent), *F*(2, 54) = 143.23, *p* < 0.001, *η*_*p*_^*2*^ = 0.84, as the flanker effect varied with prime compatibility (Fig. 5).

Given the significant interaction in the omnibus MANOVA, we continued with more focused 2 × 2 MANOVAs. The first MANOVA revealed a within-trial interaction without controlling for the RAE – that is, an interaction between prime compatibility (*compatible*, *incompatible*) and flanker congruency (congruent, incongruent) *F*(1, 55) = 265.36, *p* < 0.001, *η*_*p*_^*2*^ = 0.83 – because the flanker effect was larger in prime-compatible trials (56 ms) than in prime-incompatible trials (−3 ms). The second MANOVA revealed a RAE – that is, an interaction between prime compatibility (*compatible*, *neutral*) and flanker congruency (congruent, incongruent), *F*(1, 55) = 174.72, *p* < 0.001, *η*_*p*_^*2*^ = 0.76 – because the flanker effect was larger in prime-compatible trials (56 ms) than in prime-neutral trials (29 ms). The third MANOVA revealed a RAE-controlled within-trial interaction – that is, an interaction between prime compatibility (*neutral*,* incompatible*) and flanker congruency (congruent, incongruent), *F*(1, 55) = 108.98, *p* < 0.001, *η*_*p*_^*2*^ = 0.67 – because the flanker effect was larger in prime-neutral trials (29 ms) than in prime-incompatible trials (−3 ms).

### Mean ER

The omnibus 3 × 2 MANOVA revealed three significant effects. First, there was a main effect of prime compatibility, *F*(2, 54) = 35.89, *p* < 0.001, *η*_*p*_^*2*^ = 0.57: mean ER varied among prime-compatible (4.9%), prime-neutral (4.8%), and prime-incompatible (10.8%) trials. Second, there was a main effect of flanker congruency, *F*(1, 55) = 6.28, *p* = 0.015, *η*_*p*_^*2*^ = 0.10: mean ER was higher in flanker-incongruent trials (7.3%) than in flanker-congruent (6.3%) trials. Third, there was a two-way interaction between prime compatibility (compatible, neutral, incompatible) and flanker congruency (congruent, incongruent), *F*(2, 54) = 54.39, *p* < 0.001, *η*_*p*_^*2*^ = 0.67.

Given the interaction in the omnibus MANOVA, we conducted more focused 2 × 2 MANOVAs. The first MANOVA revealed a within-trial interaction without controlling for the RAE – that is, an interaction between prime compatibility (*compatible*,* incompatible*) and flanker congruency (congruent, incongruent), *F*(1, 55) = 110.47, *p* < 0.001, *η*_*p*_^*2*^ = 0.67 – because the flanker effect was larger in prime-compatible trials (4.1%) than in prime-incompatible trials (−2.7%). The second MANOVA revealed a RAE – that is, an interaction between prime compatibility (*compatible*,* neutral*) and flanker congruency (congruent, incongruent), *F*(1, 55) = 18.57, *p* < 0.001, *η*_*p*_^*2*^ = 0.25 – because the flanker effect was larger in prime-compatible trials (4.1%) than in prime-neutral trials (1.4%). The third MANOVA revealed a RAE-controlled within-trial interaction – that is, an interaction between prime compatibility (*neutral*,* incompatible*) and flanker congruency (congruent, incongruent), *F*(1, 55) = 37.71, *p* < 0.001, *η*_*p*_^*2*^ = 0.41 – because the flanker effect was larger in prime-neutral trials (1.4%) than in prime-incompatible trials (−2.7%).

## Discussion

Consistent with the conflict type and operating space accounts, we observed a RAE-controlled within-trial interaction. This finding is inconsistent with the RAE account, which does not predict such an interaction. Furthermore, when combined with the results of Experiment 1, the RAE-controlled interaction that we observed in Experiment 2 appears to favor the operating space account over the conflict type account. The operating space account posits that the size of the RAE-controlled within-trial interaction should vary positively with the size of the prime compatibility and/or flanker effect. Hence, this account can easily explain why the within-trial interaction was (1) small and not significant in Experiment 1 (wherein there was a relatively small prime compatibility effect) but (2) large and significant in Experiment 2 (wherein there was a relatively large prime compatibility effect). In contrast, the conflict type account has difficulty explaining this across-experiment difference. This account predicted that *both* experiments would produce a RAE-controlled within-trial interaction because both experiments included two distractors that trigger the same type of conflict (Kornblum, 1994).

### Exploratory comparisons of experiments 1 and 2

**Fig. 6:**
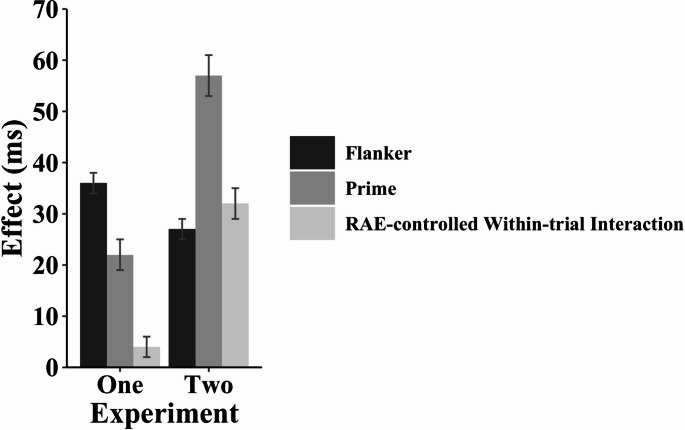
Comparison of the prime compatibility effect, flanker congruency effect, and RAE-controlled within-trial interaction in Experiments 1 and 2. Note. The size of the RAE-controlled within-trial interaction scales with the size of the prime compatibility effect in Experiments 1 and 2 but not with the size of the flanker effect. The values we plot here were calculated before rounding the group-averaged mean RTs for each condition. Therefore, these values may differ (e.g., by 1 ms) from those computed with the rounded condition means presented in the tables. Error bars indicate ± 1 standard error of the condition mean

Both the prime compatibility effect and the RAE-controlled within-trial interaction were numerically larger in Experiment 2 than in Experiment 1. These effects suggest that increasing the size of the prime compatibility effect provides more operating space for a RAE-controlled within-trial interaction to emerge in the hybrid prime-flanker task. To determine whether these effects were statistically significant, we conducted exploratory, between-subjects F-tests to contrast (1) the prime compatibility effect and (2) the interaction between prime compatibility (*neutral*,* incompatible*) and flanker congruency (congruent, incongruent) in Experiments 1 and 2 (see Fig. 6). We also contrasted the flanker effect. In total, we conducted six exploratory F-tests. Thus, after Bonferroni correction, only p-values < 0.0083 (i.e., 0.05/6) are significant.

### Mean RT

There were three significant effects. First, the prime compatibility effect was larger in Experiment 2 (57 ms) than in Experiment 1 (22 ms), *F*(1, 110) = 55.47, *p* < 0.001, *η*_*p*_^*2*^ = 0.34. Second, the RAE-controlled within-trial interaction was also larger in Experiment 2 (32 ms) than in Experiment 1 (4 ms), *F*(1, 110) = 60.25, *p* < 0.001, *η*_*p*_^*2*^ = 0.35. These findings appear consistent with the operating space account wherein the within-trial interaction requires a sufficiently large congruency effect from one or more distractors. Third, unlike the prime compatibility effect, the flanker effect was smaller – not larger – in Experiment 2 (27 ms) than in Experiment 1 (36 ms) *F*(1, 110) = 10.58, *p* = 0.002, *η*_*p*_^*2*^ = 0.09. This outcome suggests that the size of the prime compatibility effect, rather than the size of the flanker effect, determines whether there is sufficient operating space for a RAE-controlled within-trial interaction to appear in the hybrid prime-flanker task.

### Mean ER

There were three significant effects. First, the prime compatibility effect was larger in Experiment 2 (6.0%) than in Experiment 1 (2.5%), *F*(1, 110) = 14.02, *p* < 0.001, *η*_*p*_^*2*^ = 0.11. Second, the RAE-controlled within-trial interaction was also larger in Experiment 2 (4.1%) than in Experiment 1 (−0.3%), *F*(1, 110) = 23.62, *p* < 0.001, *η*_*p*_^*2*^ = 0.18. Analogous to the results of the mean RT analyses, these findings appear consistent with the operating space account wherein the within-trial interaction requires a sufficiently large congruency effect from one or more distractors. Third, unlike the prime compatibility effect, the flanker effect was smaller – not larger – in Experiment 2 (1.0%) than in Experiment 1 (4.2%), *F*(1, 110) = 34.25, *p* < 0.001, *η*_*p*_^*2*^ = 0.24. As in the mean RT analyses, this outcome suggests that the size of the prime compatibility effect, rather than the size of the flanker effect, determines whether there is sufficient operating space for a RAE-controlled within-trial interaction to appear in the hybrid prime-flanker task.

### Experiment 3

The results of Experiments 1 and 2 suggest that, consistent with the operating space account, a RAE-controlled within-trial interaction appears when the prime compatibility effect is relatively large. Since these results come from a task wherein the two distractors produce the *same* type of conflict (i.e., S-S conflict), however, it remains unclear whether they generalize to a task wherein two distractors trigger *different* types of conflict. Such an outcome would fit with the operating space account. In contrast, the absence of a RAE-controlled within-trial interaction in the presence of a large prime compatibility effect might suggest that both operating space and conflict type determine when a RAE-controlled interaction appears.

To distinguish between these possibilities in Experiment 3, we employed a hybrid prime-Simon task. In this task, the target is a red or green square that appears on the left or right side of the screen. As in Experiment 2, the prime square’s color (e.g., red) engenders S-S conflict with the target’s color (e.g., green) in prime-incompatible trials. Unlike in the hybrid prime-flanker task of Experiment 2, however, the location of the target (e.g., left) engenders S-R conflict with the location of the correct response (e.g., right) in Simon-incongruent trials.

The three accounts under investigation predict the following outcomes in Experiment 3. First, the RAE account does not predict a RAE-controlled within-trial interaction. Second, the conflict type account predicts that such an interaction is unlikely as the prime and Simon (i.e., location) distractors trigger different types of conflict (i.e., S-S and S-R conflict, respectively). Third, the operating space account predicts that a RAE-controlled within-trial interaction is likely if, as we expect, the prime compatibility effect is relatively large in Experiment 3.

## Methods

### Power analyses

Analogous to Experiments 1 and 2, we powered Experiment 3 to observe both (1) the RAE – i.e., an interaction between prime compatibility (*compatible*,* neutral*) and Simon congruency (congruent, incongruent) – and (2) a within-trial interaction that is not influenced by the RAE – i.e., an interaction between prime compatibility (*neutral*,* incompatible*) and Simon congruency (congruent, incongruent). Given the effect sizes associated with these interactions in Experiment 2 (*η*_*p*_^*2*^ = 0.76, and *η*_*p*_^*2*^ = 0.67, respectively)^8^, we calculated using G*Power 3.1.9.7 that 10 participants would be sufficient to detect each interaction with over 95% power at an alpha of 0.05. To keep the sample size consistent with those in Experiments 1 and 2, however, we decided to collect usable data from 56 participants.

### Participants

Fifty-eight undergraduates from the University of Michigan participated in Experiment 3 for course credit. None of them participated in Experiments 1 or 2. We excluded two participants who performed with less than 75% accuracy. Thus, our analyses include the data from 56 participants (19 males, 37 females; mean age, 18.9; age range 18–22), each of whom reported normal or corrected-to-normal vision and hearing.

### Stimuli and apparatus

**Fig. 7:**
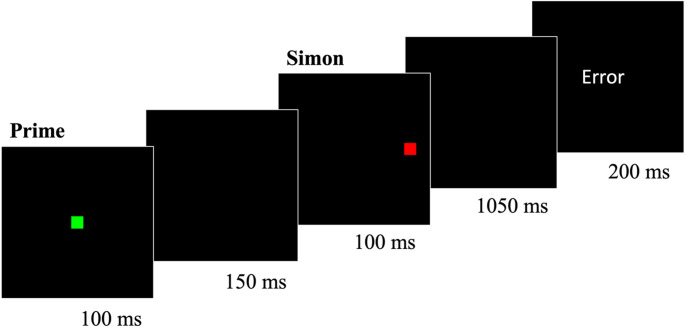
Example of a prime-Simon task trial from Experiment 3. Note. Each 1.6-s trial consisted of a prime square (colored red, green, or white) followed by a target square that appeared on either the left or right side of the screen. Participants were instructed to make a key press to identify the color (*red* or *green*) of the target square as quickly and as accurately as possible. The duration of each trial component appears beneath its respective frame

The stimuli and apparatus were identical to Experiment 2 with one exception. In Experiment 3, the target was a red or green square (~ 0.94° x ~ 0.94°) that appeared on the left or right side of the screen (~ 5.93° from center) (Fig. 7). The square’s color served as the target while the square’s location served as the Simon distractor.

### Task

The task was identical to the task in Experiment 2 with one exception: the flanker array was replaced by a single target square that appeared on the left or right side of the screen.

### Experimental design

The experimental design was the same as that in Experiment 2 with one exception: Simon congruency replaced flanker congruency. In Simon-congruent trials, the target’s location on the screen (e.g., left) matched the correct response key’s location on the computer keyboard (e.g., left). In Simon-incongruent trials, however, the target’s location on the screen (e.g., left) mismatched the correct response key’s location on the computer keyboard (e.g., right). The stimulus-response mapping (randomized across participants as in Experiments 1 and 2) determined which trials were Simon-congruent and which trials were Simon-incongruent.

### Procedure

The procedure was identical to that in Experiment 2 with one exception. In Experiment 3, we positioned the keyboards such that the midpoint between the response keys (i.e., “z” and “m”) was aligned with the center of the screen. This was to ensure that the target’s location on the screen (left or right) would correspond with the correct response key’s location on the computer keyboard (left or right) in Simon-congruent trials but not in Simon-incongruent trials.

### Data analyses

The data analyses were identical to those in Experiment 2 with one exception. In the repeated-measures MANOVAs, we replaced the “flanker congruency” factor with a “Simon congruency” factor. On average, 7.8% of all trials were errors. In the analysis of mean RT, 5.4% of the trials were outliers. In the analysis of mean ER, 5.4% of the trials were outliers. Tables 7 and 8 show each condition’s mean RT and mean ER, respectively. Table 9 presents the number of trials per condition following trial exclusions separately for mean RT and mean ER.

**Table 7:** Mean RTs, standard errors, and Simon effects (in milliseconds) in Experiment 3. The Simon effects we report in the table were calculated after rounding the group-averaged mean RTs for each condition. Therefore, these values may differ (e.g., by 1 ms) from the Simon effects that we report in the text, which we calculated before rounding the mean RTs for each condition. RT = response time; cC = prime-compatible, Simon-congruent; cI = prime-compatible, Simon-incongruent; nC = prime-neutral, Simon-congruent; nI = prime-neutral, Simon-incongruent; iC = prime-incompatible, Simon-congruent; iI = prime-incompatible, Simon-incongruent; Simon effect = incongruent–congruent. Std. Error = standard error of the mean across participants

Prime Compatibility	Simon Congruency	Mean RT	Std. Error	Simon Effect
Compatible	Congruent (cC)	424	6	30
	Incongruent (cI)	454	5	
Neutral	Congruent (nC)	464	6	19
	Incongruent (nI)	483	6	
Incompatible	Congruent (iC)	512	8	5
	Incongruent (iI)	517	8

**Table 8:** Mean ERs, standard errors, and Simon effects (in milliseconds) in Experiment 3. ER = error rate; cC = prime-compatible, Simon-congruent; cI = prime-compatible, Simon-incongruent; nC = prime-neutral, Simon-congruent; nI = prime-neutral, Simon-incongruent; iC = prime-incompatible, Simon-congruent; iI = prime-incompatible, Simon-incongruent; Simon effect = incongruent–congruent. Std. Error = standard error of the mean across participants

Prime Compatibility	Simon Congruency	Mean ER	Std. Error	Simon Effect
Compatible	Congruent (cC)	3.1%	0.3%	1.2%
	Incongruent (cI)	4.3%	0.6%	
Neutral	Congruent (nC)	4.6%	0.4%	1.0%
	Incongruent (nI)	5.6%	0.6%	
Incompatible	Congruent (iC)	8.6%	0.7%	0.9%
	Incongruent (iI)	9.5%	0.9%

**Table 9:** Mean number of trials per condition following exclusions in Experiment 3. RT = reaction time; ER = error rate; cC = prime-compatible, Simon-congruent; cI = prime-compatible, Simon-incongruent; nC = prime-neutral, Simon-congruent; nI = prime-neutral, Simon-incongruent; iC = prime-incompatible, Simon-congruent; iI = prime-incompatible, Simon-incongruent

Prime Compatibility	Simon Congruency	RT Analysis	ER Analysis
Compatible	Congruent (cC)	148	153
	Incongruent (cI)	150	157
Neutral	Congruent (nC)	146	154
	Incongruent (nI)	146	155
Incompatible	Congruent (iC)	140	154
	Incongruent (iI)	139	155

### Transparency and Openness

In our preregistration for Experiment 3, we provide the rationale for the manipulations, dependent measures, sample size, and data exclusions. Our preregistration, data analysis scripts, raw data, and task scripts are freely available on the Open Science Framework (https://osf.io/shfky/).

## Results

### Mean RT

**Fig. 8:**
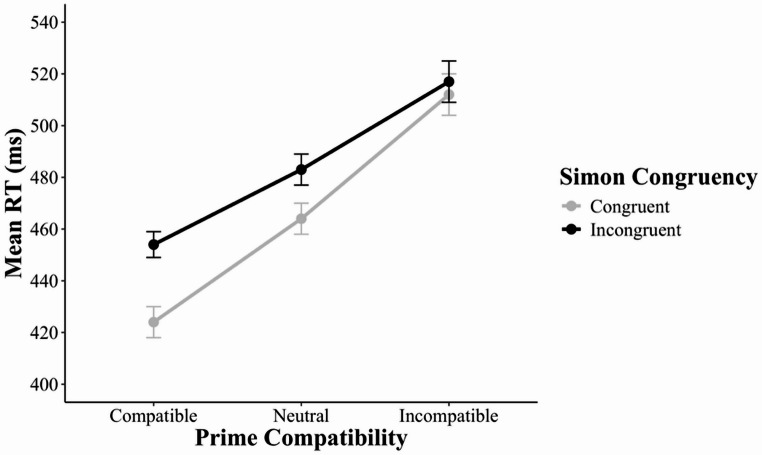
Mean reaction time as a function of prime compatibility and Simon congruency in Experiment 3. Error bars indicate ± 1 standard error of the condition mean across participants. RT = reaction time

The omnibus 3 × 2 MANOVA revealed three significant effects. First, there was a main effect of prime compatibility, *F*(2, 54) = 131.34, *p* < 0.001, *η*_*p*_^*2*^ = 0.83: mean RT varied among prime-compatible (439 ms), prime-neutral (473 ms), and prime-incompatible (514 ms) trials. Second, there was a main effect of Simon congruency, *F*(1, 55) = 57.06, *p* < 0.001, *η*_*p*_^*2*^ = 0.51: mean RT was longer in Simon-incongruent trials (484 ms) than in Simon-congruent trials (467 ms). Third, there was a two-way interaction between prime compatibility (compatible, neutral, incompatible) and Simon congruency (congruent, incongruent), *F*(2, 54) = 42.25, *p* < 0.001, *η*_*p*_^*2*^ = 0.61: the Simon effect varied with prime compatibility (Fig. 8).

Given the significant interaction in the omnibus MANOVA, we continued with more focused 2 × 2 MANOVAs. The first MANOVA revealed a within-trial interaction without controlling for the RAE – that is, an interaction between prime compatibility (*compatible*,* incompatible*) and Simon congruency (congruent, incongruent), *F*(1, 55) = 85.72, *p* < 0.001, *η*_*p*_^*2*^ = 0.61 – because the Simon effect was larger in prime-compatible trials (30 ms) than in prime-incompatible trials (4 ms). The second MANOVA revealed a RAE – that is, an interaction between prime compatibility (*compatible*,* neutral*) and Simon congruency (congruent, incongruent), *F*(1, 55) = 19.08, *p* < 0.001, *η*_*p*_^*2*^ = 0.26 – because the Simon effect was larger in prime-compatible trials (30 ms) than in prime-neutral trials (19 ms). The third MANOVA revealed a RAE-controlled within-trial interaction – that is, an interaction between prime compatibility (*neutral*,* incompatible*) and Simon congruency (congruent, incongruent), *F*(1, 55) = 33.32, *p* < 0.001, *η*_*p*_^*2*^ = 0.38 – because the Simon effect was larger in prime-neutral trials (19 ms) than in prime-incompatible trials (4 ms).

### Mean ER

The omnibus 3 × 2 MANOVA revealed two significant effects. First, there was a main effect of prime compatibility, *F*(2, 54) = 37.63, *p* < 0.001, *η*_*p*_^*2*^ = 0.58, as mean ER varied among prime-compatible (3.7%), prime-neutral (5.1%), and prime-incompatible (9.1%) trials. Second, there was a main effect of Simon congruency, *F*(1, 55) = 4.73, *p* = 0.034, *η*_*p*_^*2*^ = 0.08, as mean ER was higher in Simon-incongruent trials (6.5%) than in Simon-congruent trials (5.4%). Finally, the two-way interaction between prime compatibility (compatible, neutral, incompatible) and Simon congruency (congruent, incongruent) was not significant, *F*(2, 54) < 1. Therefore, we did not conduct additional, more focused analyses.

## Discussion

As in Experiment 2, we observed a RAE-controlled within-trial interaction. This finding is inconsistent with (1) the RAE account, which never predicts such an interaction, and (2) the conflict type account wherein S-S and S-R conflicts sum additively (Egner, 2008; Kornblum, 1994). In contrast, this finding is consistent with the operating space account because the prime compatibility effect was relatively large in Experiment 3 just as it was in Experiment 2.

Another prediction of the operating space account is that the RAE-controlled interaction in the hybrid prime-Simon task will be relatively small when the prime compatibility effect is relatively small (see Experiment 1 for an analogous result in the hybrid prime-flanker task). Although the RAE and conflict type accounts also make this prediction, for the sake of completeness we conducted a follow-up of Experiment 3 with the prime-Simon task to test this hypothesis. Here, the prime distractor was a word as in Experiment 1, rather than a color square.

Consistent with the operating space account, the mean RT data revealed a relatively small prime compatibility effect in the absence of a significant RAE-controlled within-trial interaction. Further inspection of the data, however, revealed contrasting effects in the mean RT and mean ER data. Although the Simon effect in mean RT was largest in prime-compatible trials, intermediate in prime-neutral trials, and smallest in prime-incompatible trials, the opposite pattern appeared in the mean ER data. The mean RT and mean ER data, therefore, produced contradictory results that were difficult to interpret (and may indicate the presence of speed-accuracy tradeoffs). We present this follow-up experiment in greater detail in the Appendix.

## General discussion

We investigated whether congruency effects interact in hybrid distractor-interference tasks while controlling for both contingency learning and RAE confounds. Contrary to prior findings from a hybrid prime-Simon task (Schlaghecken & Maylor, 2020), we observed a robust within-trial interaction between congruency effects in two experiments while controlling for these confounds. More specifically, we observed such an interaction when the prime compatibility effect was relatively large regardless of whether the two distractors engendered the same type of conflict or different types of conflict. These findings show for the first time that the RAE is not the sole determinant of the within-trial interaction after controlling for contingency learning confounds. In addition, these findings appear more consistent with the operating space account than with the conflict type account.

### Implications for the RAE and conflict type accounts

Schlaghecken and Maylor (2020) suggested that the RAE may explain the puzzling pattern of within-trial interactions and additive CSEs that appears frequently in hybrid distractor-interference tasks. Consistent with their suggestion, they reported that the RAE fully explained the within-trial interaction in a contingency-learning-controlled hybrid prime-Simon task. They acknowledged, however, that the RAE may not always fully explain the within-trial interaction.

The present findings confirm this possibility by showing a robust within-trial interaction when controlling for both the RAE and contingency learning confounds in (1) a hybrid prime-flanker task and (2) a hybrid prime-Simon task. This outcome is important because it suggests that processes unrelated to the RAE also contribute to the within-trial interaction in hybrid distractor-interference tasks. Thus, controlling for the RAE may not always allow researchers to distinguish between domain-specific and domain-general control (Egner, 2008; Schlaghecken & Maylor, 2020). In other words, the present findings show that controlling for the RAE may not always resolve the “generality-specificity” paradox in hybrid distractor-interference tasks.

Our findings also appear inconsistent with the conflict type account (Egner, 2008; Kornblum, 1994). Specifically, they show that the presence or absence of a RAE-controlled within-trial interaction does not vary with whether two distractors engender the same or different types of conflict even when controlling for contingency learning. This outcome adds to a growing body of work suggesting that conflict type is not the crucial variable that determines whether congruency effects interact (Rey-Mermet & Gade, 2016; Rey-Mermet, 2020; Weissman, 2020). It does not completely rule out the conflict type account, however, as we will discuss in a subsequent section.

### Implications for the operating space account

Our findings appear consistent with the operating space account: a robust RAE-controlled within-trial interaction appeared when the prime compatibility effect was relatively large but not relatively small. This outcome suggests that meaningfully interpreting the presence or absence of a within-trial interaction is possible only when there is sufficient operating space for congruency effects to interact (Hommel, 1997; Rey-Mermet, 2020). Consequently, without at least one large congruency effect, the absence of a RAE-controlled interaction may not show that (a) domain-specific control processes minimize distraction from different irrelevant stimuli (Schlaghecken & Maylor, 2020) or (b) two distractors are processed in different domains (Egner, 2008).

Why does providing more operating space increase the probability of observing a significant RAE-controlled within-trial interaction in the present tasks? One possibility stems from the view that heightened response conflict triggers an upregulation of cognitive control (Botvinick et al., 2001; Yeung et al., 2011). This view posits that when the prime compatibility (i.e., conflict) effect is relatively large, the upregulation of control that it triggers after the target appears is sufficient to influence the upregulation of cognitive control that the second (e.g., Simon) distractor triggers. Critically, this influence speeds performance when both distractors activate an incompatible response, producing a within-trial interaction (Rey-Mermet & Gade, 2016; Rey-Mermet et al., 2020). Since the upregulation of control triggered by the prime should be greater when the prime compatibility effect is relatively large (vs. small), the probability of observing a significant within-trial interaction should increase with the size of the prime compatibility effect (cf., Rey-Mermet, 2020; Weissman, 2020).

While an upregulation of control is possible, electroencephalography (EEG) data suggest some aspects of conflict processing related to two distractors can proceed independently even when a within-trial interaction occurs in mean RT. In the context of a hybrid Stroop-flanker task, for example, Rey-Mermet and colleagues (2019) reported that the Stroop and flanker effects modulate distinct event-related potential (ERP) components (i.e., the P2 and the N450, respectively) even though they interact in mean RT. Given these contradictory findings, Rey-Mermet and colleagues (2019) suggested that a within-trial interaction in mean RT may index interactions during response selection or execution rather than during earlier conflict processing (see also Egner, 2008; Xiang et al., 2024). We discuss this possibility in the next section.

### Do interactions during response selection or execution lead to within-trial interactions?

The RAE-controlled within-trial interactions that we have observed could be specific to two-choice hybrid distractor-interference tasks. In trials with two incongruent distractors, each distractor activates the same incongruent response. When the prime compatibility effect is large, coactivating the same incongruent response may lead to interactions during response selection and/or response execution that are independent of earlier conflict processing (Rey-Mermet et al., 2019). For example, such interactions may allow the incongruent response to become especially active (e.g., due to “multiplicative priming”), thereby enabling the cognitive system to more quickly identify that response as incorrect and reject it (Treccani et al., 2009, 2012; Rey-Mermet et al., 2021). A separate possibility is that when the prime strongly activates an incongruent response, the second distractor is less able to increase the activation of that response than when the prime activates its response more weakly, a different response, or no response (i.e., in neutral trials) (Xiang et al., 2024). Critically, either of these situations could reduce the congruency effect for the second distractor and thereby produce a RAE-controlled within-trial interaction.

Researchers could use 3-choice versions of the hybrid prime-flanker and prime-Simon tasks to investigate whether either of the processes above contributes to the RAE-controlled within-trial interaction. In half of the trials with two incongruent distractors, the distractors would cue the same incongruent response. In the other half, the distractors would cue different incongruent responses. In the latter half of trials, neither more quickly rejecting a highly active incongruent response nor a “ceiling” on response activation could reduce the congruency effect associated with the second (i.e., Simon or flanker) distractor. Thus, researchers could use the latter half of trials to determine whether a RAE-controlled within-trial interaction appears even while controlling for potential interactions during response selection and/or response execution.

Observing or not observing such an interaction could inform current views. First, not observing such an interaction in either task would suggest that the RAE and interactions during response selection and/or response execution can fully explain the within-trial interaction. Second, observing such an interaction in the hybrid prime-flanker task but not in the hybrid prime-Simon task would suggest a role for conflict type in producing such an interaction. Third, observing such an interaction in both tasks would suggest that other processes (e.g., interacting conflict resolution processes) contribute to this interaction. Future studies could investigate potential influences of response selection and/or response execution on the RAE-controlled within-trial interaction. Whatever the outcome, the present findings appear most consistent with the operating space account of this interaction in 2-choice tasks.

### Does parallel processing lead to within-trial interactions?

Resolving two conflicts in parallel might also explain the RAE-controlled within-trial interactions that we have observed. The time it takes to resolve two conflicts in parallel should be shorter than the sum of the times that it takes to resolve each conflict on its own (Townsend, 1990). Thus, completing two processes (e.g., two conflict resolution processes) independently and in parallel could lead to a sub-additive interaction that resembles the RAE-controlled within-trial interactions that we have observed (e.g., Risse & Oberauer, 2010).

A parallel processing view of RAE-controlled within-trial interactions makes clear predictions that can be tested in hybrid distractor-interference tasks (Weissman, 2020). First, a within-trial interaction should not appear if the prime compatibility effect is relatively large while the congruency effect associated with the second (e.g., Simon) distractor is absent. Under such conditions, there should be no opportunity for parallel processing. Second, the time it takes to resolve both conflicts should never be less than the time it takes to resolve the larger conflict. Such an outcome would suggest that resolving one conflict facilitates the resolution of the other conflict, which would argue against – rather than for – distinct conflict resolution processes that proceed independently and in parallel. Prior data from a 2-choice hybrid Stroop-Simon task are inconsistent with both predictions (Weissman, 2020). Consequently, future studies might investigate whether these predictions can be confirmed or disconfirmed in the present 2-choice tasks and/or in the hypothetical 3-choice variants of these tasks that we described earlier.

### Broader implications

The present findings are important because they indicate that the RAE appears without contingency learning confounds in both the hybrid prime-flanker and hybrid prime-Simon tasks. This outcome indicates that the RAE is a robust phenomenon that is not specific to the hybrid prime-Simon task of Schlaghecken and Maylor (2020). It also adds to prior data suggesting that the RAE stems from meta-cognitive confusion that slows performance when participants must reactivate a recently abandoned response. Consistent with this view, the RAE does not appear when participants are not conscious of the prime. This finding suggests that the RAE occurs only when participants consciously interpret conflicting response activations as contradictory (i.e., confusing) instructions (Schlaghecken & Maylor, 2020). Future studies investigating the boundary conditions for observing a RAE may provide further insights into this phenomenon.

Our findings also have important implications for recent behavioral and modeling data suggesting that distinct control mechanisms independently resolve conflicts caused by distractors appearing on three different stimulus dimensions (Gheza & Kool, 2025). These data show that the three distractors produce additive congruency effects and additive CSEs, which the authors argue is consistent with a “dimension-specific” account of control. Since the congruency effects in this recent study were relatively small, however, the present findings suggest that the additive (i.e., independent) congruency effects the authors observed may reflect a lack of operating space, rather than dimension-specific processing of different distractors. Determining whether the congruency effects in the authors’ task remain additive or interact when they are larger in size could provide a more stringent test of their “dimension-specific” account of control.

Finally, our findings may have important implications for the interpretation of recent functional MRI data (Jiang & Egner, 2014). These data suggest that mostly domain-specific but, to some extent, also domain-general neural mechanisms resolve different types of conflict in the hybrid Stroop-Simon task. To the extent that the RAE-controlled within-trial interactions we have observed also index domain-general mechanisms (Schlaghecken & Maylor, 2020), the present findings suggest that the relative proportions of domain-general and domain-specific neural mechanisms the brain recruits to resolve conflict may vary with the size of the congruency effects. In particular, the brain may recruit more domain-general neural mechanisms when at least one of the two congruency effects is relatively large but more domain-specific mechanisms when both congruency effects are relatively small. Future work could explore this possibility.

### Limitations

The present study has three main limitations. First, some of the findings supporting the operating space account come from exploratory across-experiment comparisons. Future studies could investigate whether manipulating the size of the prime compatibility effect (e.g., small, medium, or large) influences the size of the RAE-controlled within-trial interaction in a single experiment. They could also investigate whether manipulating the size of the prime compatibility effect increases the size of this interaction in a continuous or discrete manner. Whatever the outcome, the results of Experiments 2 and 3 are more consistent with the operating space account than with either the RAE account or the conflict type account. Second, if Kornblum et al.’s (1990) taxonomy of conflict types is incorrect, then the presence or absence of a within-trial interaction may depend on conflict types that differ from those in this taxonomy. We are open to this possibility, but it is unclear how an alternative taxonomy of conflict types would be organized. Third, we cannot definitively conclude that the generality-specificity paradox remains unresolved. Given our finding that congruency effects interact when the prime compatibility effect is relatively large, drawing such a conclusion would require that CSEs sum additively under the same conditions. Interpreting CSEs is difficult in the present tasks, however, because these tasks have feature integration (i.e., stimulus repetition) confounds that can influence the size of the CSE independent of putative across-trial control mechanisms (Hommel et al., 2004; Mayr, 2003). Future studies using “confound-minimized” versions of the present tasks could directly investigate whether the generality-specificity paradox remains unresolved.

## Conclusions

The present findings lead to three conclusions. First, the RAE is not the only cognitive mechanism that contributes to within-trial interactions in hybrid distractor-interference tasks. Consequently, controlling for the RAE may not always eliminate the puzzling pattern of within-trial interactions and additive CSEs that appears in such tasks. Second, the conflict type account does not accurately predict when a robust, RAE-controlled within-trial interaction appears in the present two-choice tasks. Third, consistent with the operating space account, such an interaction appears in each of these tasks when the prime compatibility effect is relatively large. Future studies investigating the generality-specificity paradox may yield other important insights into how the attentional system copes with distraction from multiple sources.

## Figures and Tables

**Fig. 9:**
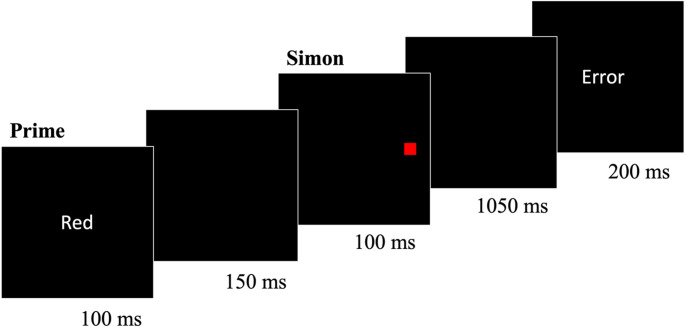
Example of a prime-Simon task trial from Experiment 4. Note. Each 1.6-s trial consisted of a prime word (“Red”, “Green”, or “Cross”) followed by a target square that appeared on either the left or right side of the screen. Participants were instructed to make a key press to identify the color (*red* or *green*) of the target square as quickly and accurately as possible. The duration of each trial component appears beneath its respective frame

**Fig. 10:**
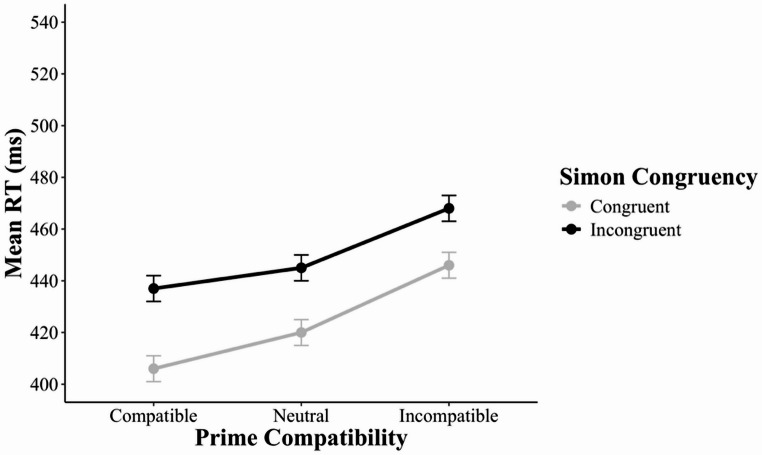
Mean reaction time as a function of prime compatibility and Simon congruency in Experiment 4. Error bars indicate ± 1 standard error of the condition mean across participants. RT = reaction time

## Data Availability

The preregistrations for Experiment 1 (https://osf.io/ux8e2/), Experiment 2 (https://osf.io/dz6qj/), Experiment 3 (https://osf.io/shfky/), and Experiment 4 (https://osf.io/xs89h/), along with the data analysis scripts, raw data, and task scripts for Experiments 1-4, are available on the Open Science Framework (OSF).

## Appendix

As we mentioned in the Discussion of Experiment 3, we conducted a fourth experiment to investigate whether a RAE-controlled within-trial interaction appears in the hybrid prime-Simon task when the prime compatibility effect is relatively small. Although this experiment produced contradictory findings in the analyses of mean RT and mean ER that we find difficult to interpret, we include this experiment in the Appendix for the sake of completeness. To reduce the size of the prime compatibility effect, we replaced the prime “color square” distractors in Experiment 3 with the words “Red”, “Green”, and “Cross” that we employed in Experiment 1. Since these prime word distractors do not share perceptual features with the target colors, they should capture attention to a lesser degree than the color squares in Experiment 3 (Buetti et al., 2014). Thus, these distractors should engender a relatively small prime compatibility effect.

All three accounts predict that a RAE-controlled within-trial interaction is unlikely in Experiment 4. By definition, the RAE account always makes this prediction. The conflict type account makes this prediction because the prime and Simon distractors trigger different types of conflict (S-S and S-R conflict, respectively). The operating space account makes this prediction if the prime compatibility effect in Experiment 4 is much smaller than in Experiment 3 as we expect. Note that only this account, which posits a role for the size of the prime compatibility effect in producing the within-trial interaction, could explain why a RAE-controlled within-trial interaction appears in the hybrid prime-Simon task when the prime compatibility effect is large (i.e., in Experiment 3) but not small (i.e., as we expect in Experiment 4).

## Methods

### Power Analyses

As in Experiments 1, 2, and 3 we powered Experiment 4 to observe two effects: (1) the RAE – i.e., an interaction between prime compatibility (*compatible, neutral*) and Simon congruency (congruent, incongruent) – and (2) a within-trial interaction that is not influenced by the RAE – i.e., an interaction between prime compatibility (*neutral, incompatible*) and Simon congruency (congruent, incongruent). Given the effect sizes associated with these interactions in Experiment 3 (*η*_*p*_^*2*^= 0.26, and *η*_*p*_^*2*^ = 0.38, respectively), we calculated in G*Power 3.1.9.7 that 41 participants would be sufficient to detect each interaction with over 95% power at an alpha of 0.05. To make the sample size consistent with those in Experiments 1-3, however, we decided to collect usable data from 56 participants.

### Participants

Sixty-two^9^ undergraduate students from the University of Michigan participated in Experiment 4 for course credit. None had participated in Experiments 1–3. We excluded six of these participants from our analyses because they performed with less than 75% accuracy. Thus, the analyses that we present for Experiment 4 only include data from the remaining 56 participants (13 males, 43 females; mean age, 19.1; age range 18–22), each of whom reported having normal or corrected-to-normal vision and hearing.

### Stimuli and Apparatus

The stimuli and apparatus were identical to those in Experiment 3 with one exception: the prime in each trial was the word “Red”,“Green”, or “Cross”, as in Experiment 1 (Fig. 9).

### Task and Experimental Design

The task and experimental design were identical to those in Experiment 3 except for the stimuli that served as prime distractors.

### Procedure

The procedure was identical to that in Experiment 3.

### Data Analyses

The data analyses were identical to those in Experiment 3. On average, 9.5% of all trials were errors. In the analysis of mean RT, 5.5% of the trials were outliers. In the analysis of mean ER, 5.5% of the trials were outliers. Tables 10 and 11 present mean RT and mean ER in each condition, respectively, following these exclusions. Table 12 presents the number of trials in each condition following trial exclusions separately for mean RT and mean ER.

**Table 10:** Mean RTs, standard errors, and Simon effects (in milliseconds) in Experiment 4. The Simon effects we report in the table were calculated after rounding the group-averaged mean RTs for each condition. Therefore, these values may differ (e.g., by 1 ms) from the Simon effects that we report in the text, which we calculated before rounding the mean RTs for each condition. RT = response time; cC = prime-compatible, Simon-congruent; cI = prime-compatible, Simon-incongruent; nC = prime-neutral, Simon-congruent; nI = prime-neutral, Simon-incongruent; iC = prime-incompatible, Simon-congruent; iI = prime-incompatible, Simon-incongruent; Simon effect = incongruent–congruent. Std. Error = standard error of the mean across participants

Prime Compatibility	Simon Congruency	Mean RT	Std. Error	Simon Effect
Compatible	Congruent (cC)	406	5	31
	Incongruent (cI)	437	5	
Neutral	Congruent (nC)	420	5	25
	Incongruent (nI)	445	5	
Incompatible	Congruent (iC)	446	5	22
	Incongruent (iI)	468	5

**Table 11:** Mean ERs, standard errors, and Simon effects in Experiment 4. ER = error rate; cC = prime-compatible, Simon-congruent; cI = prime-compatible, Simon-incongruent; nC = prime-neutral, Simon-congruent; nI = prime-neutral, Simon-incongruent; iC = prime-incompatible, Simon-congruent; iI = prime-incompatible, Simon-incongruent; Simon effect = incongruent–congruent. Std. Error = standard error of the mean across participants

Prime Compatibility	Simon Congruency	Mean ER	Std. Error	Simon Effect
Compatible	Congruent (cC)	3.4%	0.3%	3.4%
	Incongruent (cI)	6.8%	0.7%	
Neutral	Congruent (nC)	4.5%	0.4%	4.5%
	Incongruent (nI)	9.0%	0.9%	
Incompatible	Congruent (iC)	6.6%	0.5%	5.8%
	Incongruent (iI)	12.4%	1.1%

**Table 12:** Mean number of trials per condition following exclusions in Experiment 4. RT = reaction time; ER = error rate; cC = prime-compatible, Simon-congruent; cI = prime-compatible, Simon-incongruent; nC = prime-neutral, Simon-congruent; nI = prime-neutral, Simon-incongruent; iC = prime-incompatible, Simon-congruent; iI = prime-incompatible, Simon-incongruent

Prime Compatibility	Simon Congruency	RT Analysis	ER Analysis
Compatible	Congruent (cC)	143	148
	Incongruent (cI)	143	153
Neutral	Congruent (nC)	144	150
	Incongruent (nI)	139	152
Incompatible	Congruent (iC)	141	151
	Incongruent (iI)	133	152

### Transparency and Openness

In our preregistration for Experiment 4, we provide the rationale for the manipulations, dependent measures, sample size, and data exclusions. Our preregistration, data analysis scripts, raw data, and task scripts are freely available on the Open Science Framework (https://osf.io/xs89h/).

## Results

### Mean RT

The omnibus 3 × 2 MANOVA revealed three significant effects. First, there was a main effect of prime compatibility, *F*(2, 54) = 211.06, *p* < 0.001, *η*_*p*_^*2*^ = 0.89, as mean RT varied among prime-compatible (422 ms), prime-neutral (432 ms), and prime-incompatible (457 ms) trials. Second, there was a main effect of Simon congruency, *F*(1, 55) = 177.16, *p* < 0.001, *η*_*p*_^*2*^ = 0.76, as mean RT was longer in Simon-incongruent trials (450 ms) than in Simon-congruent (424 ms) trials. Third, there was a two-way interaction between prime compatibility (compatible, neutral, incompatible) and Simon congruency (congruent, incongruent), *F*(2, 54) = 9.67, *p* < 0.001, *η*_*p*_^*2*^ = 0.26, because the Simon effect varied with prime compatibility (Fig. 10).

Given the significant interaction in the omnibus MANOVA, we continued with more focused 2 × 2 MANOVAs. The first MANOVA revealed a within-trial interaction without controlling for the RAE – that is, an interaction between prime compatibility (*compatible*,* incompatible*) and Simon congruency (congruent, incongruent), *F*(1, 55) = 19.69, *p* < 0.001, *η*_*p*_^*2*^ = 0.26 – because the Simon effect was larger in prime-compatible trials (31 ms) than in prime-incompatible trials (22 ms). The second MANOVA revealed a RAE – that is, an interaction between prime compatibility (*compatible*,* neutral*) and Simon congruency (congruent, incongruent), *F*(1, 55) = 10.19, *p* = 0.002, *η*_*p*_^*2*^ = 0.16 – because the Simon effect was larger in prime-compatible trials (31 ms) than in prime-neutral trials (25 ms). The third MANOVA did not reveal a significant RAE-controlled within-trial interaction – that is, an interaction between prime compatibility (*neutral*,* incompatible*) and Simon congruency (congruent, incongruent), *F*(1, 55) = 3.03, *p* = 0.087, *η*_*p*_^*2*^ = 0.05 – because the Simon effect did not differ between prime-neutral trials (25 ms) and prime-incompatible trials (22 ms).

### Mean ER

The omnibus 3 × 2 MANOVA revealed three significant effects. First, there was a main effect of prime compatibility, *F*(2, 54) = 43.81, *p* < 0.001, *η*_*p*_^*2*^ = 0.62, as mean ER varied among prime-compatible (5.1%), prime-neutral (6.8%), and prime-incompatible (9.5%) trials. Second, there was a main effect of Simon congruency, *F*(1, 55) = 40.70, *p* < 0.001, *η*_*p*_^*2*^ = 0.43, as mean ER was higher in Simon-incongruent trials (9.4%) than in Simon-congruent trials (4.8%). Third, there was an interaction between prime compatibility (compatible, neutral, incompatible) and Simon congruency (congruent, incongruent), *F*(2, 54) = 6.44, *p* = 0.003, *η*_*p*_^*2*^ = 0.19.

Given the significant interaction in the omnibus MANOVA, we continued with more focused 2 × 2 MANOVAs. The first MANOVA revealed a within-trial interaction without controlling for the RAE – that is, an interaction between prime compatibility (*compatible*,* incompatible*) and Simon congruency (congruent, incongruent), *F*(1, 55) = 13.11, *p* < 0.001, *η*_*p*_^*2*^ = 0.19. Unexpectedly, however, the Simon effect was smaller – not larger – in prime-compatible trials (3.4%) than in prime-incompatible trials (5.8%). This outcome suggests a speed-accuracy tradeoff because we observed the opposite pattern in the mean RT data. The second MANOVA revealed a reverse RAE – as indexed by an interaction between prime compatibility (*compatible*,* neutral*) and Simon congruency (congruent, incongruent), *F*(1, 55) = 4.23, *p* = 0.044, *η*_*p*_^*2*^ = 0.07 (i.e., a RAE) – because the Simon effect was smaller – not larger – in prime-compatible trials (3.4%) than in prime-neutral trials (4.5%). This outcome suggests a speed-accuracy tradeoff because we observed the opposite pattern in the mean RT data. The third MANOVA revealed a RAE-controlled within-trial interaction – that is, an interaction between prime compatibility (*neutral*,* incompatible*) and Simon congruency (congruent, incongruent), *F*(1, 55) = 4.69, *p* = 0.035, *η*_*p*_^*2*^ = 0.08. Unexpectedly, however, the Simon effect was *larger* – not smaller – in prime-incompatible trials (5.8%) than in prime-neutral trials (4.5%). This pattern differs from the lack of a significant RAE-controlled within-trial interaction that we observed in the mean RT data.

## Discussion

Our findings in Experiment 4 are more difficult to interpret than those in Experiments 1–3. First, we observed a within-trial interaction without controlling for the RAE in the mean RT data whose direction reversed in the mean ER data, suggesting a speed-accuracy tradeoff. Second, we observed a RAE in the mean RT data whose direction also reversed in the mean ER data, once again suggesting a speed-accuracy tradeoff. Third, although we did not observe a RAE-controlled within-trial interaction in the mean RT data, we observed such an interaction in the mean ER data whose direction was *opposite* to what we expected. That is, we observed a *larger* – not smaller – Simon effect in prime-incompatible trials than in prime-neutral trials.

Nonetheless, the lack of a RAE-controlled interaction in the mean RT data appears more consistent with the operating space account than with the conflict type account. Indeed, only the former account can explain why the RAE-controlled within-trial interaction in mean RT was (a) large and significant in Experiment 3 (wherein there was a relatively large prime compatibility effect) but (b) small and not significant in Experiment 4 (wherein there was a relatively small prime compatibility effect). In contrast, the conflict type account predicted that *neither* of these experiments was likely to produce a within-trial interaction, because each experiment included two distractors that engender different types of conflict (i.e., S-S conflict and S-R conflict).

## Footnotes

1 Following Schlaghecken and Maylor (2020), we use the terms prime *compatibility* and Simon *congruency*. Thus, we will refer to a prime compatibility effect and a Simon congruency effect.

2 Note, however, that such additivity was observed only when the Simon effect was measured on the horizontal axis. When the Simon effect was measured on the vertical axis, it interacted with the prime compatibility effect in the response time data. It is difficult to interpret this interaction, however, because an interaction in the opposite direction occurred in the error rate data, suggesting a speed-accuracy tradeoff.

3 The lack of a RAE-controlled within-trial interaction in Schlaghecken & Maylor’s (2020) hybrid prime-Simon task is also consistent with the conflict type account if the prime distractor and the Simon distractor trigger completely different types of conflict. However, this possibility is unlikely. The prime distractor (e.g., a leftward-pointing arrow) triggers a stimulus-stimulus conflict with the target (e.g., a rightward-pointing arrow). In contrast, the Simon distractor (e.g., a screen location on the left) triggers both a stimulus-response conflict with the location of the response (e.g., a key on the right) and a stimulus-stimulus conflict with the target (e.g., a rightward-pointing arrow). Since the prime distractor and the Simon distractor each trigger a stimulus-stimulus conflict with the target, the conflict type account appears to predict a RAE-controlled within-trial interaction in this task.

4 We are not aware of any prior studies that reported a within-trial interaction without a speed-accuracy tradeoff while controlling for both contingency learning and the RAE. However, the partial eta squared values for within-trial interactions confounded with the RAE are often much larger than 0.20 (e.g., Weissman, 2020, Experiment 1).

5 All figures that illustrate example trials were made in Microsoft PowerPoint. All figures that present graphs of data were created using the ggplot2 package for the R programming language.

6 Responses made after the deadline were also considered omissions.

7 Note that 2 × 2 repeated-measures MANOVAs produce the same F- and p-values for main effects and interactions as corresponding 2 × 2 repeated-measures ANOVAs when there is only a single dependent measure as in the present study.

8 The effect size values for these interactions deviate slightly from those that we report in our pre-registration (*η*_*p*_^*2*^ = 0.76, *η*_*p*_^*2*^ = 0.66). This deviation is present because the group statistics for Experiment 2 listed in our Experiment 3 pre-registration include a participant that we later determined did not meet the eligibility requirements for inclusion in the study (i.e., normal hearing). Ultimately, we replaced this participant.
